# Triatomines intrahaemocoelic inoculation protocol: a useful tool to check infectivity in insects

**DOI:** 10.14440/jbm.2016.46

**Published:** 2016-05-23

**Authors:** Gabriela S. Rozas-Dennis, Gerardo A. Marti, Nadia Lorena González, Felipe Guhl, Diego M. A. Guérin

**Affiliations:** ^1^Departamento de Biología, Bioquímica y Farmacia, Universidad Nacional del Sur, Bahía Blanca, Argentina; ^2^Centro de Estudios Parasitológicos y de Vectores (CEPAVE CCT-La Plata-CONICET-UNLP), La Plata, Argentina; ^3^Departamento de Ciencias Biológicas, Centro de Investigaciones en Microbiología y Parasitología Tropical, (CIMPAT), Universidad de los Andes, Carrera ^1^E No. 18 A-10, Bloque A, Bogotá, Colombia; ^4^Departamento de Ciencias Biológicas, CIMPAT, Universidad de los Andes, Bogotá, Colombia; ^5^Departamento de Bioquímica y Biología Molecular, Facultad de Ciencia y Tecnología, Universidad del País Vasco (EHU), Instituto Biofisika (UPV/EHU, CSIC), Leioa, Vizcaya, Spain; ^§^Current address: Centre de Recherche en Ethique de la Santé - HELESI- Université Catholique de Louvain Promenade de l’Alma 51 1200 Bruxelles, Belgium.

**Keywords:** Chagas disease, inoculation protocol, intrahaemocoelic inoculation, Triatoma virus, Triatomines

## Abstract

Vectors of Chagas disease are currently controlled by employing several chemical insecticides though there is a continuing search for alternative ecological methods against disease causing vectors. An effective method includes the use of specific pathogens as biological control agents. The aim of this work was to describe a complete experimental inoculation protocol in triatomines. The intrahaemocoelic inoculation technique can be applied to inoculate different kinds of microorganisms such as viruses, fungi, bacteria and protozoa; so it could be considered a useful tool in infective bioassays. This article includes results from evaluations of *Triatoma virus* (TrV, *Dicistroviridae*: *Triatovirus*) infectivity in several triatomine species. The protocol, also suitable for any other kind of insects, describes the materials and steps required to safely inoculate the insects, preventing any damage and/or contamination.

## BACKGROUND

Triatomines are hemipterous insects with 143 species already reported [1] and known by different common names in diverse American countries (“vinchucas” in Argentina, “pito” in Colombia, “barbeiros” in Brazil, “kissing-bugs” in the United States, “chinches” in Mexico, “chipos” in Venezuela, “chirimachas” in Peru, etc.). Many species, throughout America, are well recognized as vectors of Chagas disease, an endemic zoonotic disease affecting almost 7-8 million people [2]. This disease is considered one of the most important health problems in Latin America, placed fourth in importance, behind respiratory illnesses, diarrhea and AIDS [3] and currently emerging in non-endemic countries [4].

**Figure 1.**
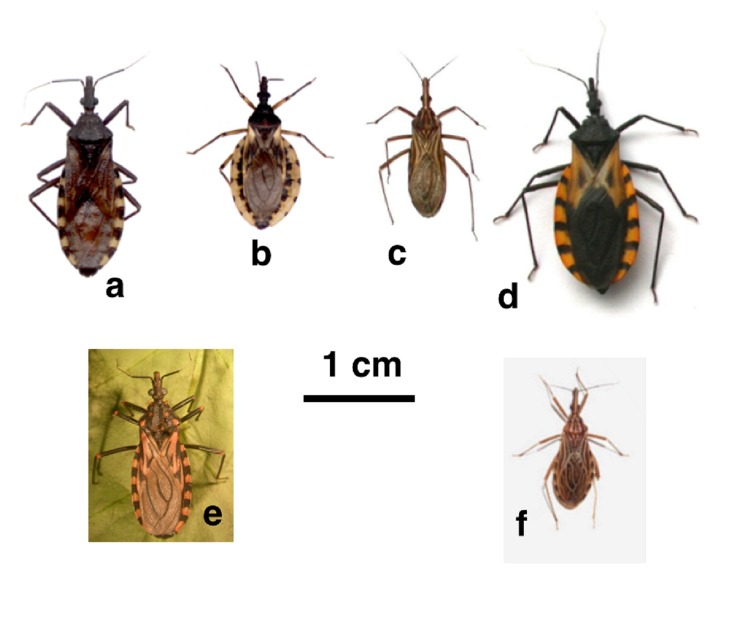
**Images of Triatomine individuals belonging to different species.** From left to right: a. *T. infestans*(♀), b. *T. guasayana* (♀)*, *c. *Rhodnius prolixus (*♂), d. *T. dimidiata*(♀), e. *T. maculata*(♀) *and* f.*R. ecuadoriensis* (♂)*. T. infestans* individuals were obtained from domestic and peridomestic populations of Bahía Blanca city, Argentina (38° 43´04´´ S y 62° 14´ 55´´O). *T. guasayana* individuals were obtained from the Centro de Referencia de Vectores of the Servicio Nacional de Chagas (CVR) insectary. Córdoba, Argentina. *T. dimidiata, T. maculata, R. ecuadoriensis and R. prolixus* were obtained from the Centro de Investigaciones en Microbiología y Parasitología Tropical, (CIMPAT) insectary. Bogotá, Colombia.**

There are 17 triatomine species reported in Argentina and although *Triatoma infestans* is the most important one due to its strictly synantropic habits, other peridomiciliary species were found naturally infected with the parasite *Trypanosoma cruzi*, transmitter of Chagas disease. Such is the case of *Triatoma guasayana* and *Triatoma patagonica* [5, 6]. Regarding triatomines in Colombia, 26 species have been reported, 15 out of them considered of epidemiological importance such as *Triatoma dimidiata, Triatoma maculata* and *Rhodnius prolixus* [7-9]. Some of the species of epidemiological importance either in Argentina and Colombia are illustrated in **Figure 1.** Since the 1980s, an alternative way to the classical chemical control of Chagas disease vectors was explored and specific pathogens as biological control agents of triatomines were investigated [10-12].

*Triatoma virus* (TrV; *Dicistroviridae: Cripavirus*), is a small virus isolated from *T. infestans* [13] which inhibits the molting process, raises mortality in nymphs instars, and reduces longevity and fecundity in adults [14-16] thus, with interesting qualities to be considered as a potential triatomine control agent.

Although this virus was naturally found in *T. infestans*, *T. sordida*, *T. delpontei* and *Psammolestes coreodes* [10,17,18] the number of susceptible species has increased to 16 after acquiring TrV infection either horizontally, by oral route (*i.e* by coprophagy) or by intrahaemocoelic inoculation. Currently, *T. infestans*, *T. platensis, T. delpontei, T. pallidipenis, T. rubrovaria, Rhodnius prolixus, R. ecuadorienses T. sordida T. patagonica, T guasayana, T. dimidiata, T. maculata, T. breyeri, Psammolestes coreodes, R. neglectus and Meccus longipennis* are cited as hosts for TrV [16-21]. The two last species were reported infected in one insectary in Brazil [19].

This report describes a step-by-step method to carry out an intra haemocoelic inoculation in triatomines. The inocula consisted of different dilutions of *Triatoma virus* (*Dicistroviridae*), extracted from infected *T. infestans* colonies reared in laboratory and the triatomine species treated were *T. infestans, T. maculata, T. dimidiata, R. ecuadoriensis, and R. prolixus.*

## MATERIALS

• A visual demonstration of the materials needed can be found in **Movie S1** (11’’ to 52’’).
• Safety material: latex gloves, facial mask, protective glasses and laboratory coat.
• For insect manipulation and readiness: pincers, entomological pins, cotton hyssop and alcohol solution (70% in distilled water)
• For insect inoculation: a 10 µl Hamilton syringe (SYR 10 µl 701N) and small hypodermic needle (approx. 15 mm long and 0.5 mm in diameter, 25 G 5/8¨)
• The inoculum: The inoculum employed in all the individuals was 0.3 µg/µl of purified TrV in NMT buffer solution and the control inoculum consisted of 3 µl of the same buffer [13]. Viral solutions were placed in 1 ml Eppendorf.
• Sealer post-inoculation: paraffin wax, burner and Pasteur pipettes.
• Elements needed post inoculation: 250 ml plastic glass and paper fan 4 cm high.

**Figure 2.**
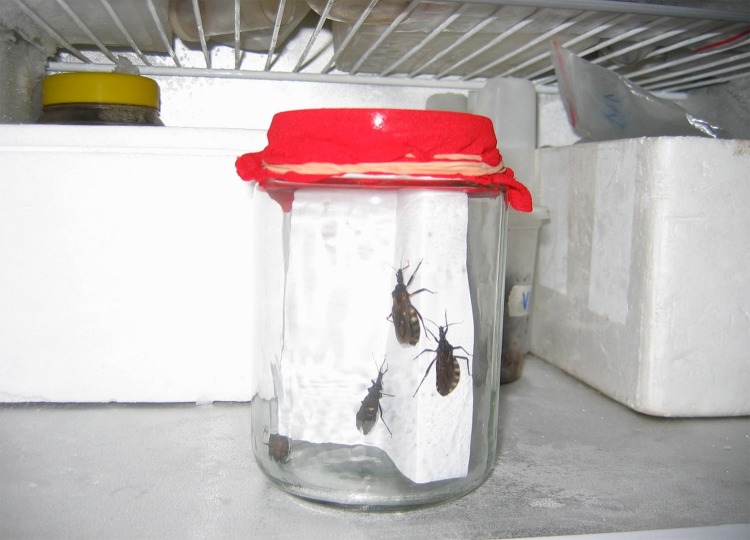
**Insect anesthetization.** Recipient containing the triatomine insects stored for about 1 min at -4ºC in order to be anesthetized before inoculation process.

## PROCEDURE

### Triatomine manipulation (Movie S1, 1’02” to 1’26”)

**Figure 3.**
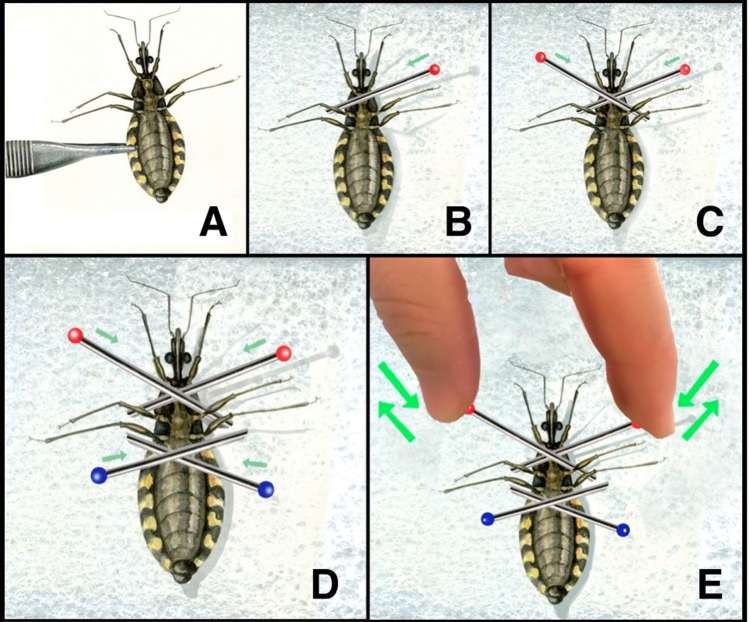
**Insect manipulation steps. A.** Gathering the insect with pincers; **B**, **C**, **D**. Insect immobilization with entomological pins; **E.** Tightening or untightening the pins.

1. Vectors manipulation frequently requires that the insect be anesthetized [23] **(Fig. 2)**. This can be achieved by placing vectors inside a recipient and leaving the insect in refrigerator for a minute.
2. Start the procedure by gathering the insect with the pincers **(Fig. 3A)** and placing its dorsal part on the cork plate.
3. Immobilize the insect by placing entomological pins crisscrossing the insect body and its coxae (**Fig. 3B-D**)
4. Be sure it´s possible to tight and untight the pins if necessary **(Fig. 3E).** Try not to hurt the insect.

### Inoculation Process (Movie S1, 1’27” to 2’17”)

5. Fill the Hamilton syringe with the inoculum
6. Disinfect the inoculation zone with alcohol solution using a cotton hyssop.
7. Take the small hypodermical needle to punch a hole in which the inoculum will be injected.
8. Hold softly the insect conexive by pincers and perforate the insect intersegmental sternal abdominal zone with the hypodermical needle. Keep the bevel upward.
9. Introduce the syringe needle a few millimeters (5–10 mm, according to the insect size, and at about the middle of the insect abdominal zone) inside the practiced hole and inject the desired solution very slowly. Then, take out the needle gently.

### Sealing process (Movie S1, 2’18” to 2’28”)

10. Immediately, cover the hole on the insect body with a single drop of warm paraffin. Be sure that the paraffin isn´t too hot.

### Post-inoculation actions (Movie S1, 2’29”to 2’43”)

11. Let the triatomine free and allow it to walk inside the plastic recipient. A paper fan can be placed to let the insect climb on it, to check that there was no mechanical damage.
12. Disinfect the syringe needle with a piece of alcohol swab.

**Figure 4.**
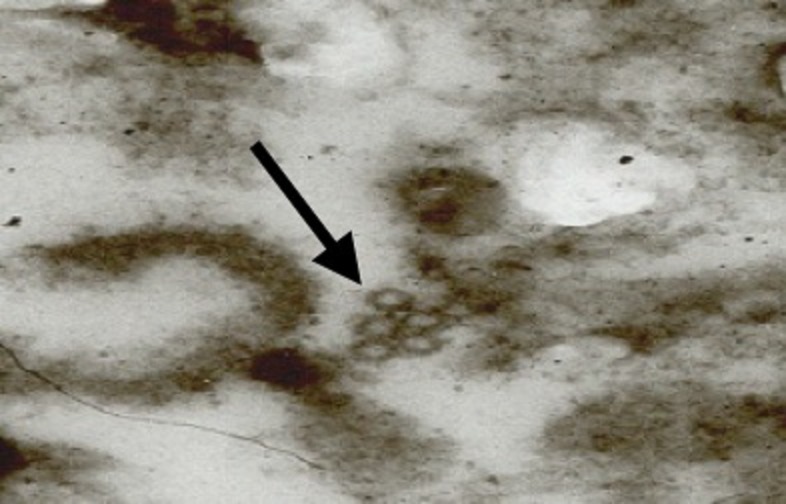
**Image of TrV particles**. Images of TrV particles in the inoculum taken by transmission electron microscopy (TEM) × 66.000. The sample consists of feaces from inoculated specimens of *R. prolixus*, obtained by the technique as described in Method 2 of reference 22. Arrows indicate TrV particles. Image: INS-CIMPAT 2007.

## ANTICIPATED RESULTS

The inoculation protocol reported here is illustrated with one experiment in which experimental infection with TrV is evaluated.

The presence of TrV particles in the inoculum was checked using transmission electron microscopy (T.E.M) [22], by observation of drops of fresh faecal material from the inoculated insects (**Fig. 4**). The treated insects were inoculated with 3 µl of purified TrV solutions obtained from infected *T. infestans* [22]. Likewise, the same number of control insects were inoculated with buffer NMT (0.01 M NaCl, 0.001 M MgCl_2_, 0.05 M Tris HCl, pH 7.5).

The corresponding results are shown in **Table 1** in which mortality rates and time of death were evaluated after inoculation. Due to the control insect deaths, and in order to correct systematic errors (most likely arising by the insect manipulation), Abbott´s formula was applied (see the following document: apps.who.int/iris/handle/10665/70070).

Abbott´s formula:


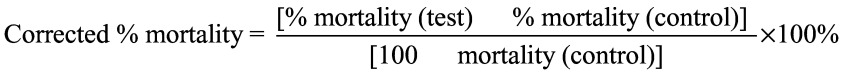


## DISCUSSION

An intrahaemocoelic inoculation protocol for triatomines is described here, and could be used with diverse kinds of insects. It has advantages such as easy, quick and economical. Previous reports about TrV inoculation showed that some of the treated insects became infected and showed symptoms of leg paralysis in its typical TrV host, *T. infestans* [10, 15] and also in *T. patagonica* [16].

To calculate the corrected experimental mortality rate, we accounted for systematic errors (*e.g.* injury caused by the injection) in the control group by applying Abbot’s formula (**Table 1**).

Based on our data and as expected, the highest mortality was observed in the species *T. infestans as expected*. The lowest mortality was observed in *R. prolixus* and *R. ecuadoriensis*. Most likely, common effects caused by TrV infection in all the species could be associated with the phylogenetic relationship [20].

**Table 1.** TrV infectivity experience.

Triatomine species	Insects inoculated TrV/Buffer	Dead insects TrV/Buffer	Infectivity	Mortality ^c^(%)
*T. infestans*	12/12	12/3	Positive	100
*T. maculata*	12/12	9/2	Positive	75.0
*T. dimidiata*	12/12	7/1	Positive	57.1
*R. ecuadoriensis*	12/12	7/2	Positive	45.0
*R. prolixus*^a^	12/12	5/1	Positive	43.5
*R prolixus*^b^	12/12	5/2	Positive	33.3

^a^Domestic species; ^b^Sylvatic species; ^c^Corrected by Abbott’s formula

## CONCLUSION

The inoculation protocol described herein provides a standard method to evaluate the effect caused by any toxic agent in different triatomine species. It could be useful to determine the infectivity and lethal effect of any enemy microorganisms of these insects. It was proven to be particularly useful for TrV, a potential control agent of Chagas disease vectors.

## TROUBLESHOOTING

Possible problems and their causes and troubleshooting solutions are listed in **Table 2**.

**Table 2.** Troubleshooting.

Step	Problem	Causes	Solution
1	Insect death	Frozen	Keep the insect inside the refrigerator for no more than a minute
9	Insect death	Insect digestive tract damaged	Introduce the syringe parallel to the body axis
9	Inoculum loss	Wrong syringe introduction	Syringe needle bevel must be upward
10	Insect death	Injured with hot paraffin	Be sure the paraffin is lukewarm

## Supplementary information

**Movie S1**. Triatomines intrahaemocoelic inoculation protocol.
